# Hypothalamic PKA regulates leptin sensitivity and adiposity

**DOI:** 10.1038/ncomms9237

**Published:** 2015-09-18

**Authors:** Linghai Yang, G. Stanley McKnight

**Affiliations:** 1 Department of Pharmacology, University of Washington School of Medicine, 1959 North East Pacific Street, Box 357280, Seattle, Washington 98195, USA

## Abstract

Mice lacking the RIIβ regulatory subunit of cyclic AMP-dependent protein kinase A (PKA) display reduced adiposity and resistance to diet-induced obesity. Here we show that RIIβ knockout (KO) mice have enhanced sensitivity to leptin’s effects on both feeding and energy metabolism. After administration of a low dose of leptin, the duration of hypothalamic JAK/STAT3 signalling is increased, resulting in enhanced POMC mRNA induction. Consistent with the extended JAK/STAT3 activation, we find that the negative feedback regulator of leptin receptor signalling, Socs3, is inhibited in the hypothalamus of RIIβ KO mice. During fasting, RIIβ–PKA is activated and this correlates with an increase in CREB phosphorylation. The increase in CREB phosphorylation is absent in the fasted RIIβ KO hypothalamus. Selective inhibition of PKA activity in AgRP neurons partially recapitulates the leanness and resistance to diet-induced obesity of RIIβ KO mice. Our findings suggest that RIIβ–PKA modulates the duration of leptin receptor signalling and therefore the magnitude of the catabolic response to leptin.

## Abstract (web summary)

Mice lacking RIIβ, a regulatory subunit of protein kinase A, are lean and resistant to diet-induced obesity. Here, the authors show that RIIβ regulates leptin sensitivity, acting as a physiological brake on leptin responsiveness and the duration of leptin signalling in the hypothalamus.

## Graphical abstract

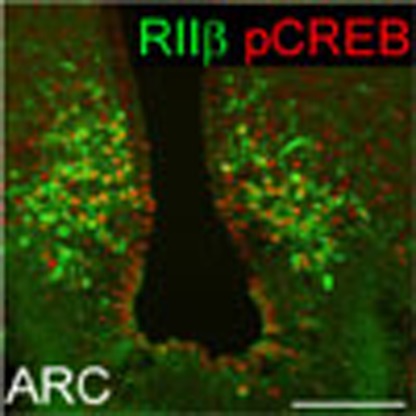

The adipose-derived hormone, leptin, regulates energy balance by binding to receptors in the hypothalamus and regulating neural circuits that suppress feeding and increase energy expenditure^1^,^2^,^3^,^4^. Plasma leptin concentration is directly correlated with the level of stored triglyceride in white adipocytes providing a negative feedback signal to the brain to help maintain body weight homeostasis. However, the chronic consumption of calories in excess of those required for daily energy expenditure leads to elevated adiposity, a proportionate increase in circulating leptin, and a decrease in the response of neurons to leptin^5^. This hypothalamic leptin resistance has limited the usefulness of leptin as a treatment for obesity. The leptin receptor (LepRb) signals primarily through stimulation of the associated JAK2 kinase which then leads to phosphorylation of STAT3 transcription factors and changes in gene transcription^6^. In addition, JAK2 triggers the PI3K pathway leading to multiple effects including activation of Akt and subsequent phosphorylation and inactivation of the transcription factor, FoxO1 (ref. 7). The intracellular mechanisms which regulate the overall sensitivity of neurons to circulating leptin are poorly understood. The protein kinase A (PKA) regulatory (R) subunit, RΙΙβ, is highly expressed in mouse brain, brown adipose tissue and white adipose tissue with limited expression elsewhere^8^,^9^,^10^,^11^. RIIβ knockout (KO) mice exhibit a 50% reduction in white adipose tissue, a four to fivefold reduction in serum leptin, and are resistant to diet-induced obesity and diabetes^10^,^12^,^13^,^14^. Previous studies have shown that deficiency of RIIβ–PKA in GABAergic hypothalamic neurons leads to the lean phenotype^15^ but it remains unclear what the intracellular signalling events are that account for this phenotype. Although reduced serum leptin is a strong anabolic signal to promote feeding and suppress energy expenditure^16^, RIIβ KO mice exhibit only a slight increase in food intake^12^,^14^ and a normal basal metabolic rate^15^ in the context of greatly reduced serum leptin. Based on these observations, we hypothesize that RIIβ–PKA may regulate energy balance by modulating leptin signalling in the hypothalamus and its deficiency may lead to sensitized responses to leptin.

PKA holoenzyme is a heterotetramer containing a dimer of two R subunits with each binding a catalytic (C) subunit. Binding of cyclic AMP (cAMP) to the R subunits leads to its conformational change and release of active C subunits. Four R subunits genes (encoding RIα, RIβ, RIIα and RIIβ) and two C subunits genes (encoding Cα and Cβ) have been identified in mouse^17^. The R subunits act as intrinsic inhibitors of the C subunits and also protect the C subunits from degradation^18^. The RIIβ–PKA holoenzyme contains a homodimer of RIIβ and two C subunits (Cα or Cβ). We have shown previously that RIIβ deficiency leads to decreased C subunits and PKA activity in adipose tissues^10^,^11^ and striatum^8^,^19^.

In this report, we show that RIIβ–PKA regulates the sensitivity of mice to the catabolic effects of leptin on feeding and energy expenditure. We demonstrate that RIIβ–PKA is abundant in LepRb-expressing neurons in the hypothalamus and becomes activated during a fast. Inhibition of PKA by expression of a dominant negative *Prkar1a* allele in AgRP neurons inhibited hypothalamic CREB phosphorylation in fasted mice and resulted in a lean phenotype and resistance to high-fat diet-induced obesity, partially mimicking the phenotype of the RIIβ KO mouse. We suggest that the cAMP/PKA pathway plays an important physiological role in modulating the gain of LepR signalling in the hypothalamus and that this results in significant effects on overall adiposity.

## Results

### Disruption of RIIβ results in increased sensitivity to leptin

**Figure 1:**
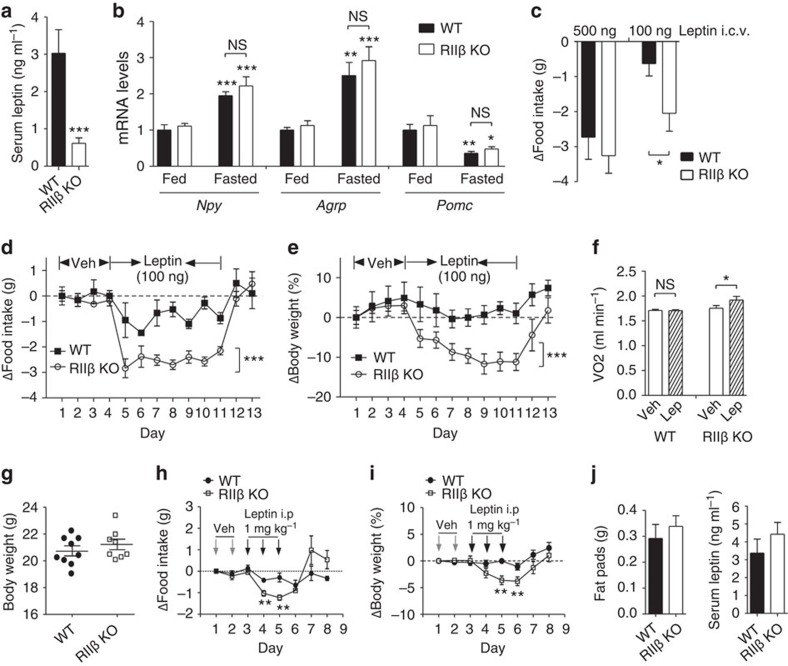
RIIβ KO mice show enhanced response to leptin. (**a**) Serum leptin levels of 12-week-old age-matched male wild-type (WT) (*n*=7) and RIIβ KO mice (*n*=9). (**b**) Hypothalamic Npy (fed WT *n*=6, KO *n*=8; fasted WT *n*=11, KO *n*=11), Agrp (fed WT *n*=5, KO *n*=5; fasted WT *n*=11, KO *n*=11) and Pomc mRNA (fed WT *n*=6, KO *n*=8; fasted WT *n*=11, KO *n*=11) from fed and fasted male WT and RIIβ KO mice. Messenger RNA values were determined by quantitative reverse transcription–PCR and standardized to LepRb mRNA. Fed WT mice were set at one in all cases. For each group of mice, *n*=5–11. (**c**) 24-h food intake change in male WT (*n*=5) and RIIβ KO mice (*n*=5) in response to 500 or 100 ng leptin i.c.v. administration into the third ventricle. (**d**) Changes in daily food intake and (**e**) body weight of male WT (*n*=7) and RIIβ KO (*n*=11) mice over 13 days following vehicle or leptin i.c.v. injections as indicated. Statistical significance was determined by analysis of variance (ANOVA). (**f**) Oxygen consumption of male WT (*n*=5) and RIIβ KO mice (*n*=8) following vehicle or leptin i.c.v. injection. (**g**–**j**) Body weight-matched (**g**) WT (20.7±0.4 g, 4-week-old, *n*=9) and RIIβ KO (21.2±0.4 g, 6-week-old, *n*=8) male mice were selected for leptin injections. (**h**) Changes in daily food intake and (**i**) body weight of WT and RIIβ KO mice over 8 days following vehicle or leptin i.p. injections as indicated. (**j**) Fat pads weight and serum leptin levels were collected and assessed after the experiments. Data are expressed as mean±s.e.m. Two-way ANOVA was used for the effect of genotypes followed by Bonferroni’s *post hoc* tests or Student’s *t*-tests (*P<0.05, **P<0.01, ***P<0.001). NS, not significant.

The mRNA levels of leptin-regulated orexigenic peptides, NPY and AgRP, and the anorexigenic αMSH precursor POMC in the hypothalamus are similar between RIIβ KO and wild-type (WT) control mice in both fed and fasted states despite the very low leptin levels (Fig. 1a,b). This indicates that RIIβ KO mice have an intact leptin-responsive system in the hypothalamus but suggests that they might be hypersensitive to leptin.

To test leptin sensitivity, we injected low or high doses of leptin directly into the third ventricle of 6–8-week-old male RIIβ KO mice and WT controls. By measuring 24-h food intake, we determined that RIIβ KO and WT littermates respond equivalently to a single injection of a high dose of leptin (500 ng). However, when the leptin dose was reduced to 100 ng, there was a greater reduction of 24-h food intake in KO mice compared with WT mice (Fig. 1c). To further examine the effect of central leptin administration on body weight loss, leptin at a dose of 100 ng was injected through a cannula into the third ventricle of KO and WT mice once a day for 7 days. Food intake was suppressed to a much greater extent in KO mice compared with control WT mice (Fig. 1d). As expected from the food intake data, this low dose of leptin caused only a slight decrease in body weight in WT mice but a very significant loss of body weight in the KO mice which was rapidly reversed within 48 h after leptin was discontinued (Fig. 1e). Energy expenditure, as measured by oxygen consumption, was increased by >10% in KO mice but not in control WT mice after 100 ng leptin infusion into the third ventricle (Fig. 1f). We considered the possibility that the increased sensitivity to leptin in the adult KO mice might simply reflect their leanness at a time when the age-matched WT mice are beginning to gain fat mass and develop leptin resistance. To test this possibility, we examined weight and adiposity-matched WT and KO mice at a young age (Fig. 1g, j). Leptin induced greater suppression of food intake (Fig. 1h) and loss of body weight (Fig. 1i) in adiposity-matched RIIβ KO mice compared with WT mice, indicating that the elevated leptin sensitivity of RIIβ KO mice is not a secondary effect of their lean phenotype.

### PKA regulates the duration of JAK/STAT signalling by LepRb

**Figure 2:**
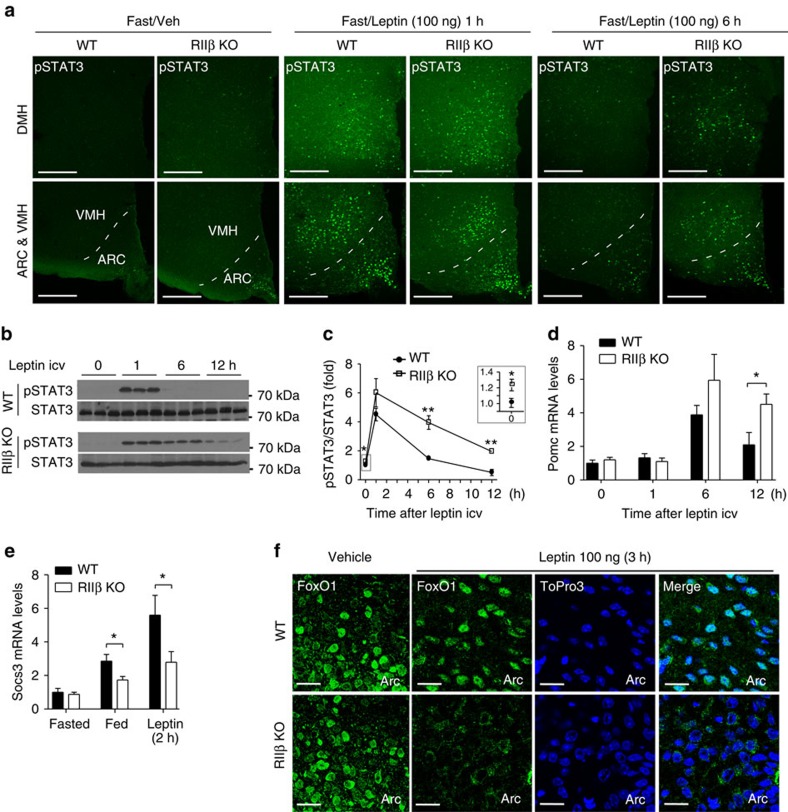
Enhanced leptin-induced STAT3 phosphorylation and POMC expression in the hypothalamus of RIIβ KO mice. (**a**) Representative images of pSTAT3 immunofluorescent staining in hypothalamic regions of fasted (24 h) WT and RIIβ KO mice (*n*=3 for each group) with vehicle or leptin i.c.v injections at different time points. pSTAT3, tyrosine^705^-phosphorylated STAT3. ARC, arcuate nucleus; VMH, ventromedial hypothalamic nucleus; DMH, dorsomedial hypothalamic nucleus. Scales bars, 200 μm. (**b**) Representative immunoblots of pSTAT3 (from five mice per group in three independent experiments) with hypothalamic lysates from WT and RIIβ KO mice at different time points after 100 ng leptin (1, 6 and 12 h) or vehicle i.c.v. injection (0 h). Each lane represents one mouse. (**c**) STAT3 phosphorylation normalized to total STAT3 levels quantified by densitometry. For each treatment group, *n*=5. Inset shows the initial difference in STAT3 phosphorylation of fasted mice. Prior to leptin treatment animals were fasted for 24 h. (**d**) Hypothalamic Pomc mRNA from WT and RIIβ KO mice treated with vehicle (0 h) or leptin (100 ng, i.c.v.). Animals (males) were fasted for 24 h prior to treatment. *N* values for WT:KO were 0 h (6:6), 1 h (4:4), 6 h (7:8) and 12 h (8:9). (**e**) Hypothalamic Socs3 mRNA for WT and RIIβ KO mice that were fasted for 24 h and then either re-fed for 6 h or treated with leptin as indicated. *N* values for WT:KO were fasted (12:12), fed (7:9) and leptin 2 h (8:8). (**f**) Representative images of FoxO1 immunostaining in the ARC of vehicle or leptin-treated (100 ng leptin i.c.v., 3 h) WT and RIIβ KO mice (*n*=3 per genotype for each treatment). RIIβ KO mice showed enhanced leptin-induced FoxO1 exclusion from the nucleus and degradation. ToPro3 was used to visualize the cell nucleus. Scale bars, 20 μm. Male mice were used in these studies. Data are presented as mean±s.e.m. and two-way analysis of variance was followed by Bonferroni’s *post hoc* test to determine significance (*P<0.05, **P<0.01.) Full blots are shown in Supplementary Fig. 3.

Leptin activates both the JAK2–STAT3 pathway and the phosphatidylinositol 3-kinase (PI3K)-Akt pathway in the hypothalamus to regulate energy balance^5^. STAT3 tyrosine phosphorylation (pSTAT3-Y705) is a downstream effect of LepRb activation of JAK2, phosphorylation of the LepRb on Y1138, and recruitment of STAT3 to the LepRb/JAK2 complex. pSTAT3-Y705 was induced to similar extents in KO and WT mice 1 h after low leptin (100 ng) administration to overnight fasted animals. However, the pSTAT3 signal was sustained at least 6 h longer in KO mice compared with WT mice (Fig. 2a–c). At 6 h after 100 ng leptin administration, pSTAT3-positive neurons were much more abundant in KO hypothalamus (arcuate nucleus (ARC), ventromedial hypothalamic nucleus (VMH) and dorsomedial hypothalamic nucleus (DMH)) compared with WT (Fig. 2a). Interestingly, we also observed a small but significant increase in basal hypothalamic pSTAT3 signal in overnight fasted RIIβ KO mice compared with WT control (Fig. 2a,c inset). Consistent with the prolonged leptin-induced pSTAT3 signal, the level of POMC mRNA induced by low dose leptin administration was significantly greater in KO mice than in WT mice 12 h after leptin injection (Fig. 2d).

Socs3 acts as a feedback inhibitor of the JAK/STAT pathway by inhibiting JAK2 activity^5^. Hypothalamic Socs3 mRNA levels were comparable between RIIβ KO and WT mice after an overnight fast, but were significantly lower in fed RIIβ KO mice compared with WT control (Fig. 2e). The induction of Socs3 mRNA by low dose leptin was also attenuated in RIIβ KO mice (Fig. 2e). This indicates that RIIβ–PKA deficiency may impair hypothalamic Socs3 expression and prevent the feedback inhibition that normally limits the duration of leptin signalling.

### Leptin activation of PI3K/Akt pathway is regulated by PKA

**Figure 3:**
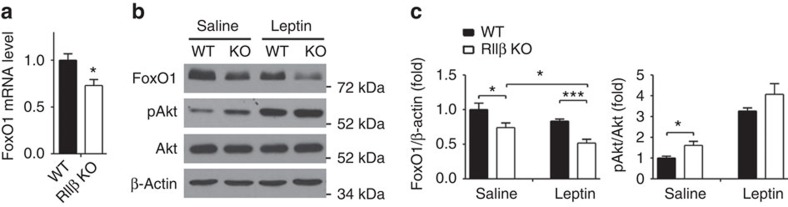
Decreased FoxO1 level in the hypothalamus of RIIβ KO mice. (**a**) mRNA levels of FoxO1 in the hypothalamus of WT and RIIβ KO mice (WT, *n*=14; RIIβ KO, *n*=17, all male). (**b**) Representative immunoblots of hypothalamic FoxO1 and pAkt (Thr308) in fasted (24 h) WT and RIIβ KO mice after saline or leptin injection. Saline or leptin (1 mg kg^−1^ body weight) was injected i.p. 30 min before mice were killed and the hypothalami were dissected and processed for western blot. (**c**) Quantification of protein levels of FoxO1 and pAkt by normalization to β-actin and total Akt, respectively. *N* values for WT:KO mice were FoxO1 saline (5:5), leptin (4:5) and pAkt/Akt saline (5:5). Values represent mean±s.e.m. *P<0.05, ***P<0.001 by Student’s *t*-tests. Full blots are shown in Supplementary Fig. 3.

Leptin also activates the PI3K/Akt pathway in several regions of the hypothalamus including the ARC triggering Akt-dependent phosphorylation and inhibition of the transcription factor, FoxO1. FoxO1 binds to the *Pomc* promoter and antagonizes the action of STAT3 leading us to ask whether this leptin receptor signalling pathway was also enhanced in RIIβ KO mice. Phosphorylation of FoxO1 by Akt triggers nuclear exclusion and proteosomal degradation of phospho-FoxO1 (refs 20, 21). To explore the effect of low leptin on ARC FoxO1 localization we administered leptin to fasted animals at 100 ng per mouse, i.c.v. and examined FoxO1 by immunohistochemistry. In fasted animals, FoxO1 was predominantly nuclear in the ARC in both WT and KO. Three hours after leptin treatment, FoxO1 remained nuclear in WT ARC neurons but became diffusely associated with the cytoplasm in many of the ARC neurons of RIIβ KO mice (Fig. 2f). We measured hypothalamic FoxO1 mRNA and protein levels and found that both were significantly decreased in the hypothalamus of RIIβ KO mice compared with WT controls (Fig. 3a–c). A higher basal level of activated Akt (phospho-Akt (T^308^)) was observed in fasted RIIβ KO hypothalamus compared with WT control and after leptin stimulation hypothalamic pAkt was markedly increased in both WT and RIIβ KO mice as expected (Fig. 3b,c). Taken together, these data indicate that RIIβ KO mice have an increased response to leptin through both the JAK2/STAT3 and Akt/FoxO1 signalling pathways in the hypothalamus.

### Regulation of LepRb signalling by PKA is cell autonomous

**Figure 4:**
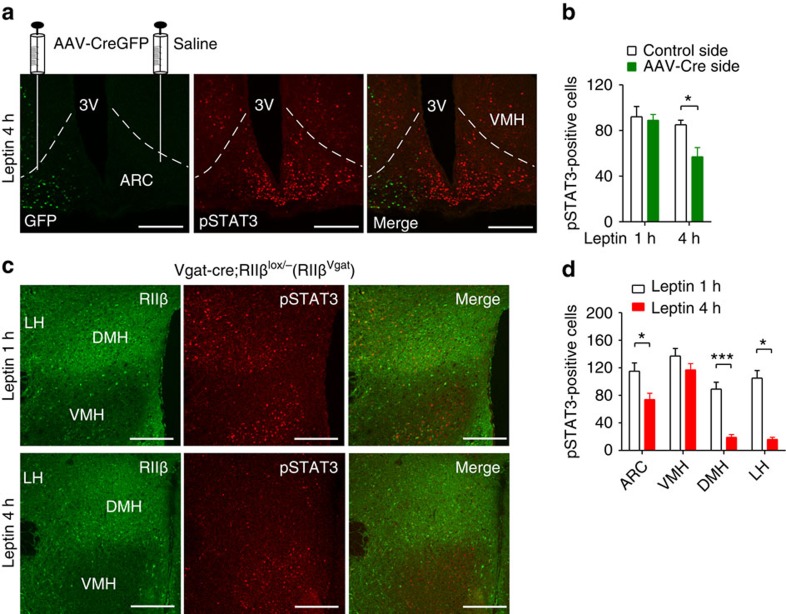
RIIβ re-expression in the hypothalamus reverses the prolonged leptin-induced pSTAT3 signalling. (**a**) Representative images (two or more sections from two to three mice) of CreGFP fluorescence and pSTAT3 immunofluorescent staining in the ventral hypothalamus from RIIβ^lox/lox^ mice injected with AAV1-CreGFP unilaterally and 4 h following leptin i.p. injection (1 mg kg^−1^). Both CreGFP and pSTAT3 were localized to cell nucleus. 3V, third ventricle; ARC, arcuate nucleus; VMH, ventromedial hypothalamic nucleus. Scale bars, 200 μm. (**b**) Quantification of pSTAT3-positive cells in the ARC side with AAV-CreGFP infection and in the saline-injected control side from mice 1 and 4 h following leptin i.p. injection (1 mg kg^−1^). Multiple sections from two mice (1 h) and three mice (4 h) were counted and averaged, and presented as cells per section. (**c**) Representative images of RIIβ and pSTAT3 immunofluroscent staining in the hypothalamus from RIIβ^Vgat^ mice 1 and 4 h following leptin i.p. injection (1 mg kg^−1^). Scale bars, 200 μm. (**d**) Quantification of pSTAT3-positive cells in hypothalamic regions ARC, VMH, DMH and lateral hypothalamus (LH) from RIIβ^Vgat^ mice 1 and 4 h following leptin i.p. injection as shown in **c** and Supplementary Fig. 1e. Sections from three mice per group were counted and averaged and presented as cells per section. Data were presented as mean±s.e.m. and analysed by two-tail Student’s *t*-test (*P<0.05, ***P<0.001).

The increased duration of STAT3 and FoxO1 signalling in RIIβ KO hypothalamus after leptin administration is occurring in multiple regions of the hypothalamus including the ARC, DMH, lateral hypothalamus (LH) and VMH. We used two approaches to ask whether the RIIβ–PKA regulation of leptin sensitivity is cell autonomous. We injected AAV-Cre into one side of the ventral hypothalamus of RIIβ^lox/lox^ mice to activate RIIβ expression (Fig. 4a and Supplementary Fig. 1a,b). STAT3 phosphorylation in the ARC was similar between the AAV-Cre-injected side and the saline-injected control side at 1 h after leptin injection (Fig. 4b) and many cells showed co-expression of Cre and pSTAT3 (Supplementary Fig. 1a), indicating that Cre expression and Cre-induced RIIβ re-expression did not affect the acute response to leptin. However, at 4 h after leptin injection, STAT3 phosphorylation was decreased in Cre-expressing cells but still robustly present in cells without Cre expression (Fig. 4a,b). As a second approach, we activated RIIβ re-expression in all GABAergic neurons by crossing the RIIβ^lox/−^ mice to a Vgat-Cre transgenic line^22^, which activated RIIβ expression in multiple hypothalamic nuclei except the primarily glutamatergic paraventricular nucleus (PVN), VMH^15^ and non-GABAergic neuronal populations in the ARC (Supplementary Fig. 1c). As shown previously, the Vgat-Cre/RIIβ^lox/−^ (RIIβ^Vgat^) mice are rescued to WT adiposity and leptin levels^15^. In the ARC, DMH and LH of RIIβ^Vgat^ mice, RIIβ was re-expressed in most leptin-responsive neurons as indicated by co-localization with leptin-induced pSTAT3 expression (Supplementary Fig. 1d). At 1 h after leptin injection (1 mg kg^−1^, i.p.) to the RIIβ^Vgat^ mice, pSTAT3 staining was evident in all the hypothalamic regions. However, at 4 h after leptin injection, most of the pSTAT3 signals disappeared in the DMH and LH but remained high in the VMH where RIIβ was not re-expressed (Fig. 4c,d). In the ARC, the number of pSTAT3-positive cells was significantly decreased at 4 h after leptin but there were still a significant number of pSTAT3-positive cells (Fig. 4d) but many of them were negative for RIIβ staining and therefore not expressing Vgat-Cre (Supplementary Fig. 1e). Our data demonstrates that RIIβ−PKA negatively regulates the duration of leptin-induced pSTAT3 signalling in hypothalamic neurons and that the enhanced leptin sensitivity in RIIβ KO neurons is cell autonomous and likely the cause rather than the effect of the lean phenotype.

### RIIβ–PKA is activated during a fast

**Figure 5:**
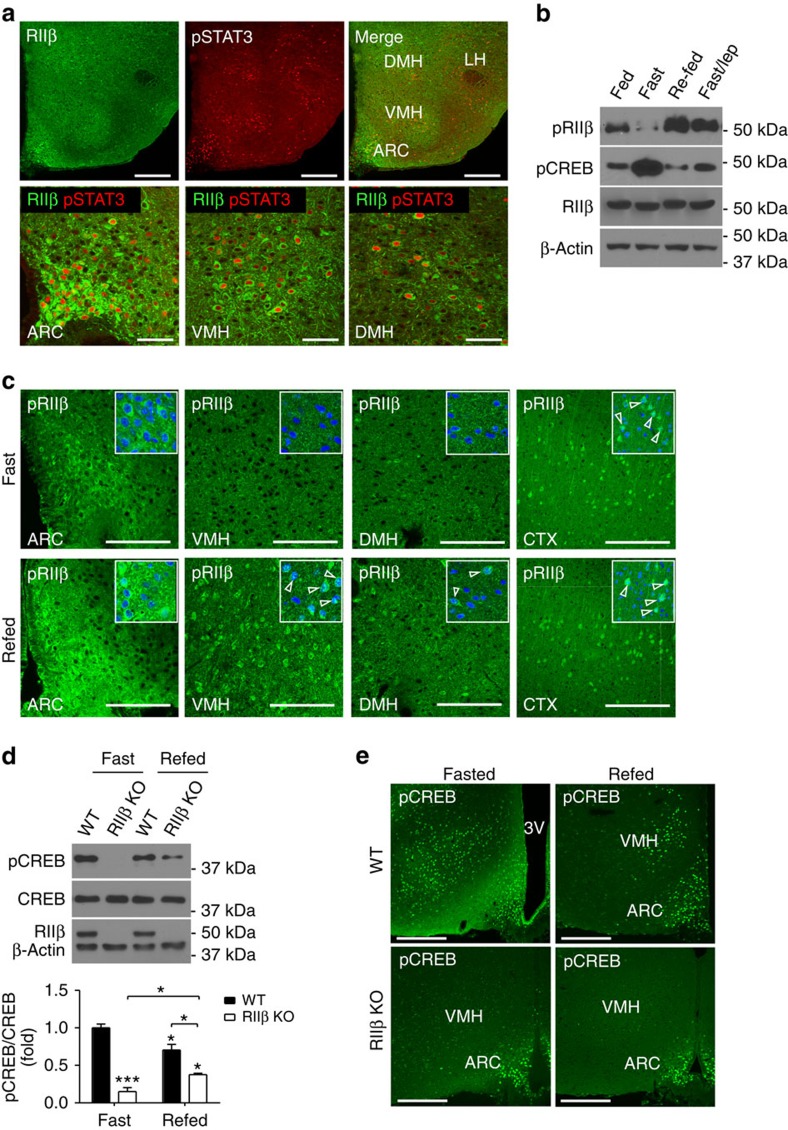
Hypothalamic RIIβ–PKA is regulated by feeding states and leptin. (**a**) Representative images of multiple sections from three mice showing co-immunofluorescent staining of RIIβ and pSTAT3 in the hypothalamus of WT mice treated with leptin (1 h after leptin i.p. 2.0 mg kg^−1^). RIIβ is localized in soma while pSTAT3 is localized in cell nucleus. Scales bars, 200 μm (upper panel) and 50 μm (lower panel). (**b**) Representative western blots (experiment repeated 3 times) of whole hypothalamic pRIIβ (S114), pCREB (S133), RIIβ and β-actin from fed, 24-h fast, 24-h fast+2-h re-feeding and 24-h fast+2-h leptin treatment (3 mg kg^−1^ BW, i.p.) of WT mice. (**c**) Representative images of pRIIβ immunofluorescnet staining in hypothalamic regions of 24-h fasted WT mice (*n*=3) with or without 2-h re-feeding. ARC, arcuate nucleus; VMH, ventromedial hypothalamic nucleus; DMH, dorsomedial hypothalamic nucleus. pRIIβ immunostaining in the cortex (CTX) was shown as internal control. Insets show the co-staining of neurons for pRIIβ and a marker for nuclear DNA (ToPro3) as indicated by arrowheads. Scale bars, 100 μm. (**d**) Representative western blots and the quantification of hypothalamic pCREB from fed, 24 h fasted, and 2 h re-fed WT and RIIβ KO mice. Total CREB, RIIβ and β-actin were blotted as internal control (*n*=3 for each condition and genotype). Data are presented as mean±s.e.m. and analysed by two-tailed Student’s *t*-test (*P<0.05, ***P<0.001 ). (**e**) Representative immunofluorescent staining of pCREB (S133) in the hypothalamus of 24-h fasted WT and RIIβ KO mice with or without 2-h re-feeding (three animals for each condition and genotype). Scale bars, 200 μm. Full blots are shown in Supplementary Fig. 3.

To determine if RIIβ–PKA is physiologically responding to nutritional signals in leptin receptor-expressing neurons as might be expected if it is regulating leptin sensitivity, we examined hypothalamic RIIβ localization and activation in fed and fasted WT mice. After a 1-h leptin treatment, pSTAT3-positive neurons were shown by immunohistochemistry to be co-expressing RIIβ (Fig. 5a). It has been suggested that RIIβ autophosphorylation at Ser114 occurs in the inactive PKA holoenzyme but does not lead to dissociation of the R/C complex^23^. Consistent with this observation, we found that activation of PKA in hypothalamic extracts by the cAMP analogue, 8-Br-cAMP, resulted in dephosphorylation of P-Ser_114_ on RIIβ and this was blocked by phosphatase inhibitors (Supplementary Fig. 2a). This allows us to directly monitor the nutritional activity of RIIβ–PKA by western blot and immunohistochemistry with pRIIβ^S114^ indicating inactive PKA and loss of pRIIβ ^S114^ as an indication of cAMP activation. As shown in Fig. 5b, a 24-h fast leads to the depletion of pRIIβ^S114^ while 2-h re-feeding greatly increases pRIIβ^S114^, suggesting that hypothalamic RIIβ–PKA is activated during fasting and inactivated after re-feeding. The fast caused an increase in pCREB^S133^ compared with fed or re-fed states (Fig. 5b), consistent with previous findings^24^. Immunostaining of pRIIβ^S114^ showed that it was increased in multiple hypothalamic nuclei including the ARC, VMH and DMH in re-fed mice compared with fasted mice (Fig. 5c). As an internal control, the level of pRIIβ^S114^ in the cortex is not changed during fasting and re-feeding (Fig. 5c). We also tested whether leptin could substitute for re-feeding and reverse the activation state of RIIβ–PKA; we were surprised to find that a 2-h treatment with leptin mimicked re-feeding and caused an increase in pRIIβ^S114^ and returned pCREB to fed levels (Fig. 5b). The specificity of anti-RIIβ and anti-pRIIβ antibodies for immunohistochemical staining was verified by staining of brain sections from RIIβ KO mice (Supplementary Fig. 2b,c). We conclude that the RIIβ–PKA holoenzyme is actively expressed in leptin-responsive neurons and is activated by fasting and inhibited by either re-feeding or leptin treatment.

### RIIβ–PKA regulates CREB phosphorylation during fasting

The level of pCREB was greatly reduced in the RIIβ KO compared with age-matched WT under either fasting or re-fed states as determined by western blot (Fig. 5d) and immuohistochemistry (Fig. 5e). To show that this effect is independent of body fat content, we examined hypothalamic pCREB levels using WT (6-week old, body weight: 20.9±0.51 g) and RIIβ KO (12-week old, body weight: 23±0.5 g) mice that had similar fat content as indicated by gonadal fat pads weight. As shown in Supplementary Fig. 2d, pCREB levels were dramatically reduced in RIIβ KO mice in either fed, fasted or re-fed state compared with fat-matched WT control. In RIIβ^Vgat^ mice, which had similar fat content and blood leptin level as age-matched WT mice^15^, pCREB expression in response to fasting was decreased in the VMH where the Vgat-Cre is not expressed and therefore RIIβ is not re-expressed. In contrast, ARC neurons exhibited both RIIβ and pCREB staining in fasted RIIβ^Vgat^ mice (Supplementary Fig. 2e). The co-localization of RIIβ and pCREB in hypothalamic neurons was shown in the ARC and VMH of fasted WT mice (Supplementary Fig. 2f). These results indicate that the phosphorylation of CREB in response to fasting is dependent on RIIβ–PKA in a cell-autonomous manner and that pCREB signalling in VMH and other non-GABAergic neurons does not play a major role in the lean phenotype of RIIβ KO mice. Consistent with the greatly attenuated pCREB expression, the protein levels of PKA C subunits (including Cα, Cβ1 and Cβ2) (Supplementary Fig. 2g) and total PKA activity (Supplementary Fig. 2h) were significantly decreased in the hypothalamus of RIIβ KO mice, indicating that RIIβ is one of the major PKA isoforms in mouse hypothalamus.

### Inhibition of PKA in AgRP neurons causes leanness

**Figure 6:**
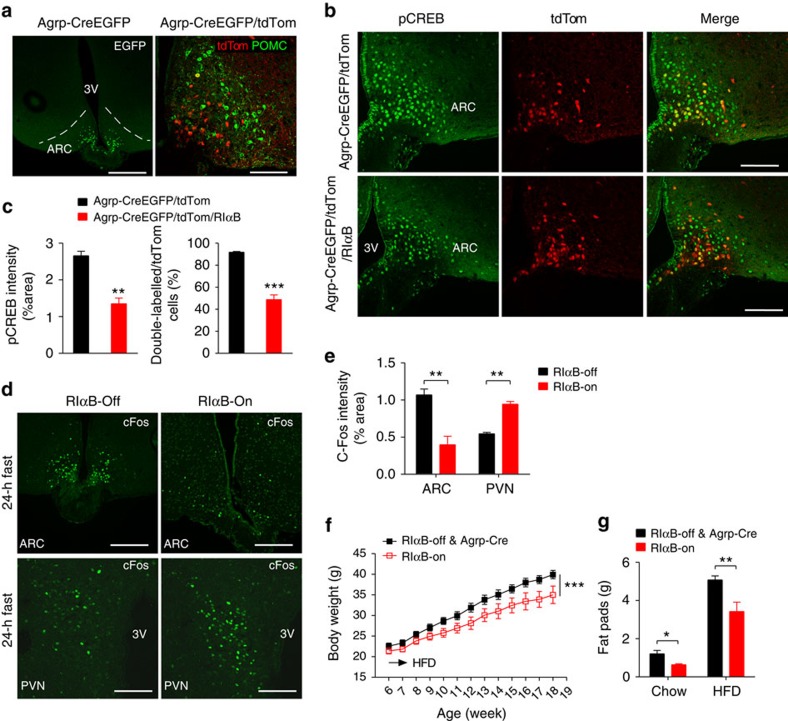
Mice with selective inhibition of PKA in AgRP neurons have reduced adiposity. (**a**) EGFP-labelled Cre expression in the hypothalamus of Agrp-CreEGFP mice (left: scale bar, 400 μm) and tdTomato expression and immunofluorescent staining of POMC in the ARC of Agrp-Cre/tdTom mice (right: scale bar, 100 μm). (**b**) Representative images of pCREB(S133) immunostaining in the ARC of Agrp-Cre/tdTom and Agrp-Cre/tdTom/RIαB mice following a 24-h fast. Scale bars, 100 μm, *n*=3 mice for each genotype (**c**) Quantification of pCREB staining intensity (left) and ratio of AgRP neurons (as indicated by tdTomato) that express pCREB (right) in the ARC of Agrp-Cre/tdTom and Agrp-Cre/tdTom/RIαB mice as shown in **b**. *N*=3 mice for each genotype. (**P<0.01, ***P<0.001 by two-tailed Student’s *t*-tests). (**d,e**) Representative immunohistochemistry (**d**) and quantification (**e**) of c-Fos expression in RIαB-Off (left) and RIαB-ON (right) mice after a 24-h fast in the ARC (scale bars, 200 μm) and PVN (scale bars, 100 μm) of the hypothalamus. *N*=3 for each genotype. (**f**) Body weight changes of Argp-Cre and RIαB-Off mice (*n*=9) and RIαB-On mice (*n*=6) on high-fat diet (HFD) from 6 week of age for 12 weeks. ***P<0.001 between genotypes by two-way analysis of variance. (**g**) Weight of major fat pads (gonadal, retroperitoneal and inguinal) of Argp-Cre mice, RIαB-Off mice and RIαB-On mice fed either on chow diet (*n*=5 per group) or HFD (as shown in **f**) at 18–19 weeks of age. Data are presented as mean±s.e.m. and analysed by two-tailed Student’s *t*-test (*P<0.05, **P<0.01, ***P<0.001).

AgRP neurons in the arcuate nucleus represent only a fraction of the GABAergic neurons in the hypothalamus but they play an essential role in the regulation of energy balance in adult mice^22^,^25^. We next asked whether impaired PKA signalling in AgRP neurons would have an effect on body composition. We generated mice with selective expression of a dominant negative PKA subunit allele (RIαB) in AgRP neurons. The dominant negative RIαB allele was generated as a knock-in mutation in *Prkar1a* that was silenced by a lox-flanked intragenic neo-stop sequence^26^,^27^. Expression of RIαB protein in AgRP neurons was initiated by Cre recombinase-dependent excision of the neo-stop sequence in Agrp-CreEGFP-expressing mice^28^. These Agrp-CreEGFP/RIαB mice are referred to as RIαB-On. The localization of Cre expression in the ARC was visualized by the fluorescence of EGFP fused to the Cre (Fig. 6a, left). Agrp-CreEGFP-mediated recombination has been shown to be specific to AgRP neurons in the hypothalamus^28^. By crossing the Agrp-Cre mouse to a tdTomato reporter mouse line^29^, we could observe Cre-activated tdTomato expression in AgRP but not in adjacent POMC neurons (Fig. 6a, right). RIαB expression significantly suppressed 24-h fasting-induced CREB phosphorylation in AgRP neurons (Fig. 6b,c). Activation of GABAergic AgRP neurons promotes feeding and inhibits energy expenditure at least partially by inhibiting the activity of PVN neurons that express melanocortin receptors^25^,^30^. We observed decreased c-Fos-positive cells in the ARC in RIαB-On mice compared with RIαB-Off mice following a 24-h fast (Fig. 6d,e). In contrast, we observed a 1.6-fold increase in c-Fos-positive cells in the PVN of fasted RIαB-On mice (Fig. 6d,e). These results indicated that PKA inhibition in AgRP neurons partially suppressed their activation by fasting and led to increased activity in a subset of PVN neurons. We examined whether RIαB-On mice recapitulated the obesity-resistant phenotype of RIIβ KO mice^12^ by placing them on a high-fat diet for 12 weeks. RIαB-On mice gained significantly less weight on a high-fat diet compared with RIαB-Off and Agrp-Cre/WT mice (Fig. 6f). Furthermore, RIαB-On mice had a significant decrease in fat pad weight compared with RIαB-Off and Agrp-Cre/WT mice on either chow or high-fat diet (Fig. 6g). These results demonstrate that PKA inhibition in AgRP neurons partially mimics the leanness of RIIβ KO mice. However, our previous studies demonstrate that re-expression of RIIβ only in AgRP neurons did not reverse the lean phenotype of RIIβ KO mice^15^, suggesting that normal PKA signalling in AgRP neurons is required but not sufficient to keep adiposity at WT level.

## Discussion

**Figure 7:**
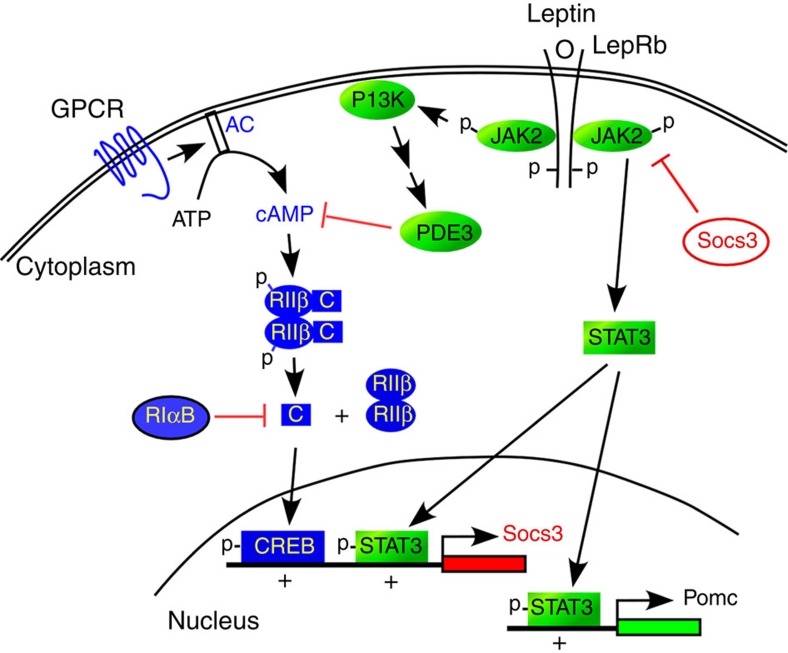
Model depicting the crosstalk between PKA and leptin signalling in hypothalamic neurons. During a fast, PKA is activated by nutritional signals acting through an unidentified Gs-coupled GPCR pathway. PKA activation leads to phosphorylation of CREB, which promotes Socs3 transcription when PSTAT3 is present. In the fed state, leptin is increased and stimulates the phosphorylation of STAT3. Leptin may also stimulate the activation of a phosphodiesterase leading to the degradation of cAMP. PSTAT3 and PCREB synergistically induce Socs3 transcription and production of the negative regulator of JAK2 kinase activity. RIIβ−PKA deficiency or expression of the dominant negative PKA allele, RIαB, disrupts basal and fasting-induced CREB phosphorylation inhibiting Socs3 induction and enhancing leptin signalling in the hypothalamus.

In this report, we show that RIIβ–PKA is a major PKA isoform in the LepR-expressing neurons of the hypothalamus and is being regulated by fasting and re-feeding. RIIβ deficiency leads to impaired PKA signalling, inhibition of pCREB induction during a fast and an increased duration of LepRb signalling through both the pSTAT3 and FoxO1 pathways in the hypothalamus. The extended duration of LepRb signalling in the RIIβ KO hypothalamus leads to an increase in the catabolic effects of low doses of leptin on feeding, energy expenditure and body weight. The ability of leptin to induce a major negative feedback regulator of LepRb signalling, Socs3, is inhibited in the RIIβ KO. An attractive hypothesis is that cAMP activation of RIIβ–PKA plays a synergistic role in the induction of Socs3 by pSTAT3, perhaps by phosphorylation of CREB (Fig. 7). The Socs3 promoter contains CRE elements that bind CREB^31^ and Socs3 can be induced in hypothalamic cell lines by cAMP^32^,^33^. Furthermore, the leanness and elevated leptin sensitivity of RIIβ KO mice resembles the phenotype of mice with either a neuron-specific KO of Socs3 (ref. 34) or global haploinsufficiency of Socs3 (ref. 35). This defective CREB signalling might also contribute to the decreased transcription of FoxO1 in RIIβ KO mice (Fig. 3a) since a recent study showed that PKA/CREB/p300 signalling promoted the expression of FoxO1 (ref. 36). The increase in leptin sensitivity we see in the RIIβ KO hypothalamus occurs in all regions where the LepRb is expressed including the ARC, VMH, DMH and LH. We have shown previously that re-expressing RIIβ in just the AgRP or POMC neurons of the ARC is not sufficient to reverse the lean phenotype. However re-expression of RIIβ selectively in GABAergic neurons with Vgat-Cre does reverse the lean phenotype^15^ and yet the glutamatergic neurons in the VMH remain deficient in RIIβ and continue to be hypersensitive to leptin (Fig. 4). In the ARC of RIIβ^Vgat^ mice, a significant number of neurons show prolonged leptin-induced pSTAT3 activation and are negative for RIIβ staining (Fig. 4d and Supplementary Fig. 1d). These RIIβ-negative neurons are likely to be the leptin-responsive glutamatergic POMC neurons^22^ that would not be expected to re-express RIIβ in the RIIβ^Vgat^ animals. These observations demonstrate that PKA signalling interacts with LepRb signalling in a cell-autonomous manner. We have not yet been able to identify the specific subset of GABAergic neurons in the hypothalamus that are responsible for the effects of RIIβ disruption on body weight regulation. It seems likely that these neurons are the same as those primary leptin-responsive neurons, still unidentified, which account for the majority of the effects of leptin on feeding and energy metabolism.

Although GABAergic neurons regulate the majority of leptin’s effects on body weight, leptin signalling in other neuronal types such as glutamatergic neurons in the VMH also contributes to the regulation of body weight^37^ and other metabolic processes including glucose homeostasis^37^,^38^ and bone metabolism^39^. We showed previously that RIIβ–PKA re-expression in SF1-expressing VMH neurons does not significantly restores the adiposity of RIIβ KO mice^15^. However, it is possible that RIIβ–PKA in the VMH might affect the susceptibility to diet-induced obesity or glucose homeostasis^12^. In addition to its role in leptin signalling, hypothalamic PKA is also a downstream effector of other metabolic signals such as glucagon like peptide (GLP1) and glucagon, which are implicated in the neuronal regulation of hepatic glucose production^40^. It is likely that RIIβ–PKA is playing other roles in overall metabolic regulation in addition to its effects on leptin signalling and adiposity focused in this report.

Recently it was reported that cAMP promotes leptin resistance by activating the Epac/Rap1 signalling pathway and increasing Socs3 expression. These authors provided evidence that the pharmacological inhibition of PKA with H89 did not prevent the forskolin-induced increase in Socs3 mRNA and protein^41^. Our studies used mouse genetic approaches to inhibit the PKA pathway and this led to an increase in leptin sensitivity and resistance to diet-induced obesity. It is likely that both PKA and Epac/Rap1 signalling pathways are being co-regulated during fasting and re-feeding as cAMP levels change in response to metabolic signals and perhaps the relative contribution of the two cAMP effector pathways depends on both the level of cAMP and the intracellular localization of the cAMP effector.

Elevated leptin sensitivity would be expected to lead to a decrease in adiposity and circulating leptin just as we see in the RIIβ KO mice. The elevated leptin sensitivity of RIIβ KO mice appears to be maintained for the animal’s lifetime and their lean phenotype may underlie the increased healthy lifespan of RIIβ KO mice^13^. The nutritional regulators that interact with hypothalamic neurons and regulate the levels of cAMP are unknown. It has been suggested that leptin itself might be capable of affecting cAMP by acting through the Akt pathway to activate PDE3 and cause a decrease in cAMP^42^. It is probable that there are also Gs-coupled GPCR pathways that stimulate adenylyl cyclase activity in LepRb-expressing neurons. A more detailed understanding of the crosstalk between the cAMP and LepRb signalling pathways may suggest a strategy for increasing leptin sensitivity therapeutically.

## Methods

### Mice

RIIβ^lox/lox^ and RIIβ^Vgat^ mice were generated and characterized as described previously^15^. We have deposited the RIIβ^lox/lox^ mice in the Mutant Mouse Regional Resource Center (MMRRC) Stock No. 036960-UCD and the RIIβ^Vgat^ mice were generated by crossing the RIIβ^lox/lox^ mice with Vgat-Ires-Cre mice sent to us by Brad Lowell (Harvard University). Agrp-CreEGFP mice were provided by Richard Palmiter (University of Washington). Conditional RIαB mice were described previously^26^,^27^ and are available as Stock No. 032879-UCD from the MMRRC. All strains were on a C57Bl/6 background. The mice were fed standard chow (Picolab mouse diet 20) or high-fat diet (Research Diets #D12492) and had free access to water. Mice were housed at 22–24 °C with a 12-h light/dark cycle and were individually housed for studies of food intake, energy expenditure and, after implantation of cannulas, for stereotactic injections. Otherwise, mice were group housed (two to five animals per cage). The number of mice used in each experiment was chosen based on the expected variance of the measurements to be used and a power analysis that would allow detection of at least a 30% change in values with a *P*<0.05. All procedures were approved by the Institutional Animal Care and Use Committee of the School of Medicine of the University of Washington.

### Leptin administration

Recombinant mouse leptin was obtained from Dr Parlow (Harbor-UCLA Medical Center, CA). Age-matched male WT and RIIβ KO mice (8–10-week old) were anesthetized and implanted with a cannula (Plastics One) into the third ventricle at the midline coordinates of 0.5 mm posterior to the bregma and 3.0 mm below the surface of the skull. After cannulation, mice were individually housed with free access to food and water, and body weight and *ad libitum* food intake were measured daily. After at least 1 week recovery from the surgery, 1 μl vehicle (artificial cerebrospinal fluid) with or without leptin was injected through the cannula into the third ventricle once a day between 1700 to 1900 hours (0–2 h before the start of dark phase). Daily food intake and body weight were measured at the time of injections. For oxygen consumption, mice were injected i.c.v. with vehicle or leptin at 1200 to 1300 hours and, 20 min later, put in the Oxymax metabolic chamber (Columbus Instruments) at room temperature for VO2 measurements for 2 h. The chamber was 4 × 8 inches, allowing limited locomotion, and the entire apparatus was housed in an isolated room away from other animals and stimuli. Air flow to the cage was 500 ml min^−1^ as previously described^43^. For each mouse, vehicle and leptin were injected on different days and VO2 was compared. Body weight-matched male WT and RIIβ KO mice were used for leptin sensitivity study with i.p. injections between 1700 to 1900 hours. The mice were killed after the experiments and the major fat pads including gonadal, inguinal and retroperitoneal fat pads were isolated and weighed; the blood was collected for serum leptin assay.

### AAV-Cre-mediated RIIβ expression

Recombinant adeno-associated virus (AAV1-CreGFP) was kindly provided by Richard Palmiter (the University of Washington). Male RIIβ^lox/lox^ mice at 8 weeks of age were anesthetized and used for Cre virus injection (1 × 10^9^ genomic particles per μl per site) into the ventral hypothalamus (1.4 mm posterior to the bregma, 0.5 mm lateral to the midline, 5.8 mm below the bregma) with a 10 μl Hamilton syringe attached to a Micro4 Micro Syringe Pump Controller (World Precision Instruments). After injection, the mouse was housed individually with free access to food and water. At least 10 days after AAV injection, mice were fasted overnight and leptin was injected (1 mg kg^−1^, i.p.). Mice were killed and perfused for pSTAT3 immunofluorescent staining at 1 or 4 h after the injection. AAV-Cre-induced RIIβ expression was determined by immunohistochemistry.

### Quantitative reverse transcription–PCR assay

We isolated total RNA from the hypothalamus using Trizol (Invitrogen). RNA concentration was determined by RiboGreen assay. SYBR Green PCR Master Mix was used for quantitative reverse transcription–PCR. LepRb mRNA level was determined using the primers shown in Supplementary Table 1 and characterized previously^44^. The data were normalized to either β-actin or *Gapdh*. No difference was observed between LepRb mRNA levels in WT and RIIβ KO mice. mRNA levels of Agrp, Npy, Pomc, Socs3 and FoxO1 were all normalized to LepRb mRNA content and expressed as a percentage change from corresponding WT control. Primers for Agrp, Npy, Pomc, β-actin, Socs3 and FoxO1 are shown in Supplementary Table 1.

### Western blot and immunohistochemistry

Western blots and immunohistochemical staining were performed as described previously^15^. Briefly, mice were killed by CO_2_. The hypothalamus (wet weight∼10 mg) was quickly dissected and snap-frozen in liquid nitrogen and saved at −80 °C for later processing. The tissue was homogenized in 200 μl lysis buffer (50 mM Tris-Cl, pH7.4, 150 mM NaCl, 5 mM EDTA, 1% Triton X-100, 0.5% deoxycholic acid) supplemented with protease and phosphatase inhibitors (5 mM NaF, 1 mM Na_3_VO_4_, PhosStop (Roche), cOmplete Ultra protease inhibitors (Roche)), sonicated and cleared by centrifugation (12,000*g*, 4 °C, 10 min). Protein concentration in the supernatant was determined by BCA assay (Pierce; 23227). About 10–20 μg of protein in 1 × SDS sample buffer (62.5 mM Tris·Cl (pH 6.8), 2% (wt/vol) SDS/5% glycerol/0.05% (wt/vol) bromophenol blue) was loaded per lane and separated by 10% SDS–PAGE and transferred to nitrocellulose membrane by electrophoresis for further blotting. For *in vitro* PKA activation, the WT hypothalamus was homogenized in 200 μl lysis buffer (50 mM Tris-HCl, pH 7.4, 150 mM NaCl, 1% Triton X-100, cOmplete Ultra protease inhibitors) and cleared by centrifugation. The supernatants were incubated at 37 °C for 10 min with or without 8-Br-cAMP (150 μM), phosphodiesterase inhibitor IBMX (3-isobutyl-1-methylxanthine; 1 mM) and protein phosphatase inhibitors (2 × PhosStop). The samples were then mixed with 2 × SDS sample buffer and heated (95 °C, 5 min) for SDS–PAGE and anti-pRIIβ and anti-pCREB immunoblotting. For immunohistochemistry, adult mice were anesthetized with pentobarbital and transcardially perfused with PBS followed by ice-cold PBS-buffered 4% paraformaldehyde. Brains were removed and postfixed for 2 h followed by cryopreservation in 30% sucrose solution (w/v) overnight and subsequent freezing in OCT compound (Tissue-Tek). Cryosections (20 μm) were taken on a cryostat and allowed to air dry on slides, followed by immediate processing or preservation at −80 °C. The total PKA activity in hypothalamic extracts of WT and RIIβ KO mice was measured using Kemptide (Sigma) as a substrate and ^32^P-γ-ATP as the phosphate donor as described previously^27^. Briefly, PKA assays were performed in duplicate using 10 μl aliquots of each protein sample (1 mg ml^−1^) and 25 μl of reaction mix (20 mM Tris buffer (pH 7.4), 200 μM ATP, 10 mM MgAcetate, 20 μM Kemptide, 0.5 mM IBMX, 10 mM dithiothreitol, 5 mM sodium fluoride and 0.045 mCi ml^−1 32^P-γ-ATP. The reaction proceeded for 5 min at 30 °C and was stopped by spotting a 25 μl aliquot onto phosphocellulose paper, which was immersed immediately in ice-cold 75 mM phosphoric acid. The filters were washed four times with phosphoric acid at room temperature, immersed in 95% ethanol and air-dried. The dried filters were placed in scintillation vials with ScintVerse (Fisher, Fair Lawn, NJ) and ^32^P-incorporation was quantified using a Beckman scintillation counter.

For pSTAT3 immunostaining, a 15-min pretreatment of the sections with 1% NaOH is required. Quantification of blots and staining intensity was obtained using ImageJ. Primary antibodies used in the study were: anti-pRIIβ (S114) (BD Biosciences, #612550); anti-RIIβ (BD Biosciences, #610626); anti-pSTAT3 (Y705) (Cell Signaling, #9131); anti-STAT3 (Cell Signaling #9132); anti-FoxO1 (Cell Signaling, #2880); anti-β-actin (Sigma, #A5316); anti-pAkt (Cell Signaling, #9275) and anti-Akt (Cell Signaling, #9272); anti-RIα (BD Biosciences #610610), anti-RI(α+β) (BD Biosciences #610165), anti-RIIα (Santa Cruz, sc-909), anti-Cα (provided by Susan Taylor, UCSD), anti-Cβ (provided by Bjorn Skalhegg, the University of Oslo), anti-pCREB (S133) (Cell Signaling, #9198), anti-CREB (Cell Signaling, #9197) and anti-cFos (Santa Cruz, sc-7202).

### Leptin measurements

Mice were killed with CO_2_, and whole blood was collected by cardiac puncture. The blood was allowed to clot at room temperature and centrifuged at 12,000 r.p.m. for 2 min. The supernatant serum was collected and saved at −80 °C for leptin assay by ELISA following the manufacturer’s protocol (Millipore, Cat# EZML-82 K).

### Statistical analyses

Data are expressed as mean±s.e.m. GraphPad Prism was used for analysis of variance with Bonferroni’s *post hoc* test or two-tailed Student’s *t*-test. *P*<0.05 was considered significant.

## Additional information

**How to cite this article:** Yang, L. and McKnight, G. S. Hypothalamic PKA regulates leptin sensitivity and adiposity. *Nat. Commun.* 6:8237 doi: 10.1038/ncomms9237 (2015).

## Supplementary Material

Supplementary Information Supplementary Figures 1-3 and Supplementary Table 1
