# Supplementary material for: Hypothalamic PKA regulates leptin sensitivity and adiposity
Source: Nat Commun. 2015 Sep 18;6:8237. doi: 10.1038/ncomms9237 (PMC4576457; doi:10.1038/ncomms9237)
Supplement: Supplementary Information — Supplementary Figures 1-3 and Supplementary Table 1 [file ncomms9237-s1.pdf]

Supplementary Figure 1 RIIβ re-expression in the hypothalamus reverses the prolonged leptin-induced pSTAT3 signaling.

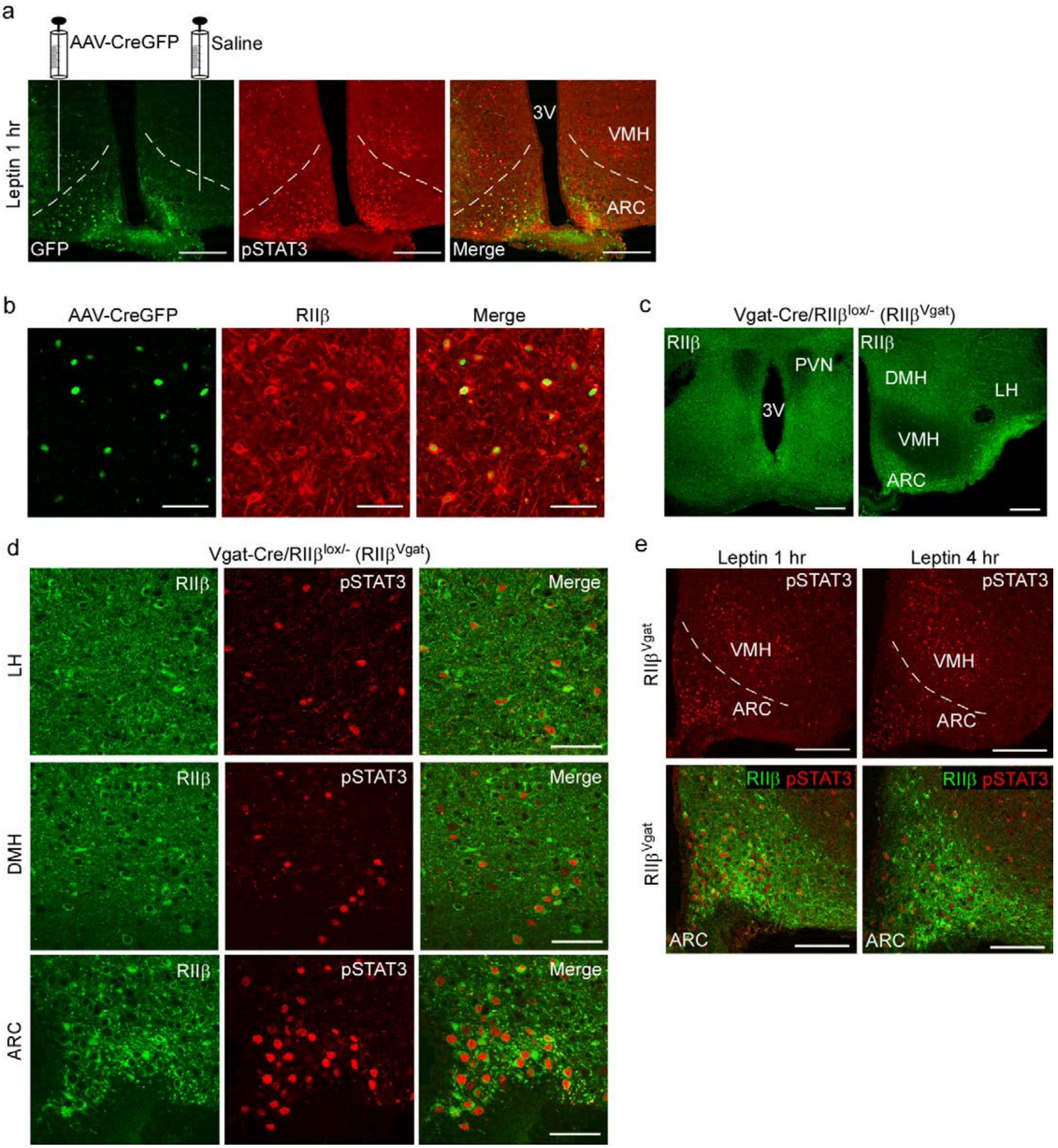

Supplementary Figure 1 RIIβ re-expression in the hypothalamus reverses the prolonged leptin-induced pSTAT3 signaling. (**a**) Representative images of CreGFP and pSTAT3 immunofluorescent staining in the ventral hypothalamus from RIIβ$^{lox/lox}$ mice (n = 3) injected with AAV1-CreGFP unilaterally and 1 h following leptin i.p. injection (1 mg/kg). Both CreGFP and pSTAT3 were localized to cell nucleus. Scale bars, 200 μm. (**b**) Representative images of CreGFP fluorescence and RIIβ immunofluorescent staining in the hypothalamus from RIIβ$^{lox/lox}$ mice with AAV1-CreGFP injection. CreGFP was localized in the nucleus while Cre-activated RIIβ expression was localized in the soma and neuronal projections. Scale bars, 50 μm. (**c**) Images of RIIβ immunofluorescent staining in the hypothalamus from RIIβ^{Vgat} mice. Scale bars, 200 μm. (**d**) Representative images of RIIβ and pSTAT3 immunofluorescent staining in the ARC, DMH and LH of 3 RIIβ^{Vgat} mice 1 h following leptin i.p injection (1 mg/kg). Scale bars, 50 μm.(**e**) Representative images of RIIβ and pSTAT3 immunofluorescent staining in the ARC and VMH of RIIβ^{Vgat} mice 1 h (n =3) and 4 h (n = 3) following leptin i.p. injection (1 mg/kg). Scale bars, 200 μm (upper panels) and 100 μm (lower panels). 3V, third ventricle; ARC, arcuate nucleus; VMH, ventromedial hypothalamic nucleus; LH, lateral hypothalamus.

Supplementary Figure 2 PKA activity and CREB phosphorylation are impaired in the hypothalamus by RIIβ disruption.

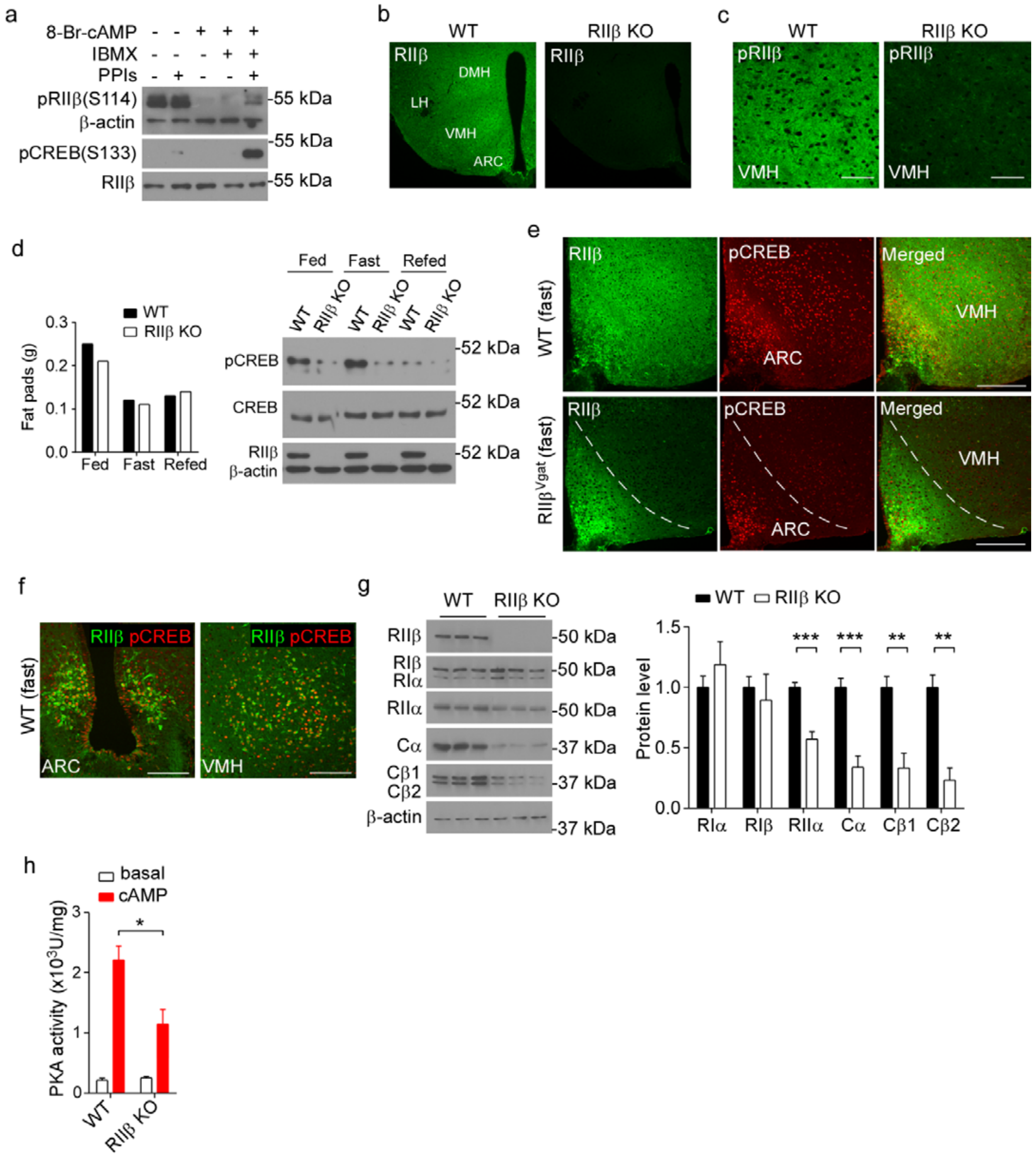

Supplementary Figure 2 PKA activity and CREB phosphorylation are impaired in the hypothalamus by RIIβ disruption. (**a**) Western blots of pRIIβ and pCREB in hypothalamic extracts incubated at 37°C for 5 min with or without cAMP analog 8-Br-cAMP (150 μM) to activate PKA. Phosphodiesterase inhibitor IBMX (1mM) and 2× phosphatase inhibitors (PPIs) (PhosSTOP, Roche) were added as indicated. (**b,c**) Representative images of immunofluorescent staining of RIIβ (**b**) and pRIIβ (**c**) in the hypothalamus of fasted WT (n = 3) and RIIβ KO (n = 3) mice. Scale bars, 50 μm. (**d**) Western blots of pCREB with hypothalamic samples from fat-matched WT (4 week old) and RIIβ KO (6 week old) male mice fed ad libitum (fed), 24 h fast (fast), and 24 h fast followed by 2 h refeeding (refed). (**e**) Representative immunofluorescent staining of pCREB in the hypothalamus of WT (n = 3) and $RII\beta^{Vgat}$ (n = 3) mice after 24 h fasting. Scale bars, 200 μm. (**f**) Co-immunofluorescent staining of RIIβ and pCREB in the hypothalamus of WT following a 24-h fast. Scale bars, 100 μm. ARC, arcuate nucleus; VMH, ventromedial hypothalamic nucleus. (**g**) Representative Western blots (left) and quantification (right) of PKA subunits including RIα, RIβ, RIIα, Cα, Cβ1 and Cβ2 in the hypothalamus of WT and RIIβ KO mice (n = 5 per group). Each lane of the blots represents a single mouse. (**h**) Basal and 5 μM cAMP-stimulated PKA activity in the hypothalamic lysates from WT and RIIβ KO mice (n = 3 per group). Full blots are shown in Supplementary Figure 3.

Supplementary Figure 3 Full scans of Western blots.

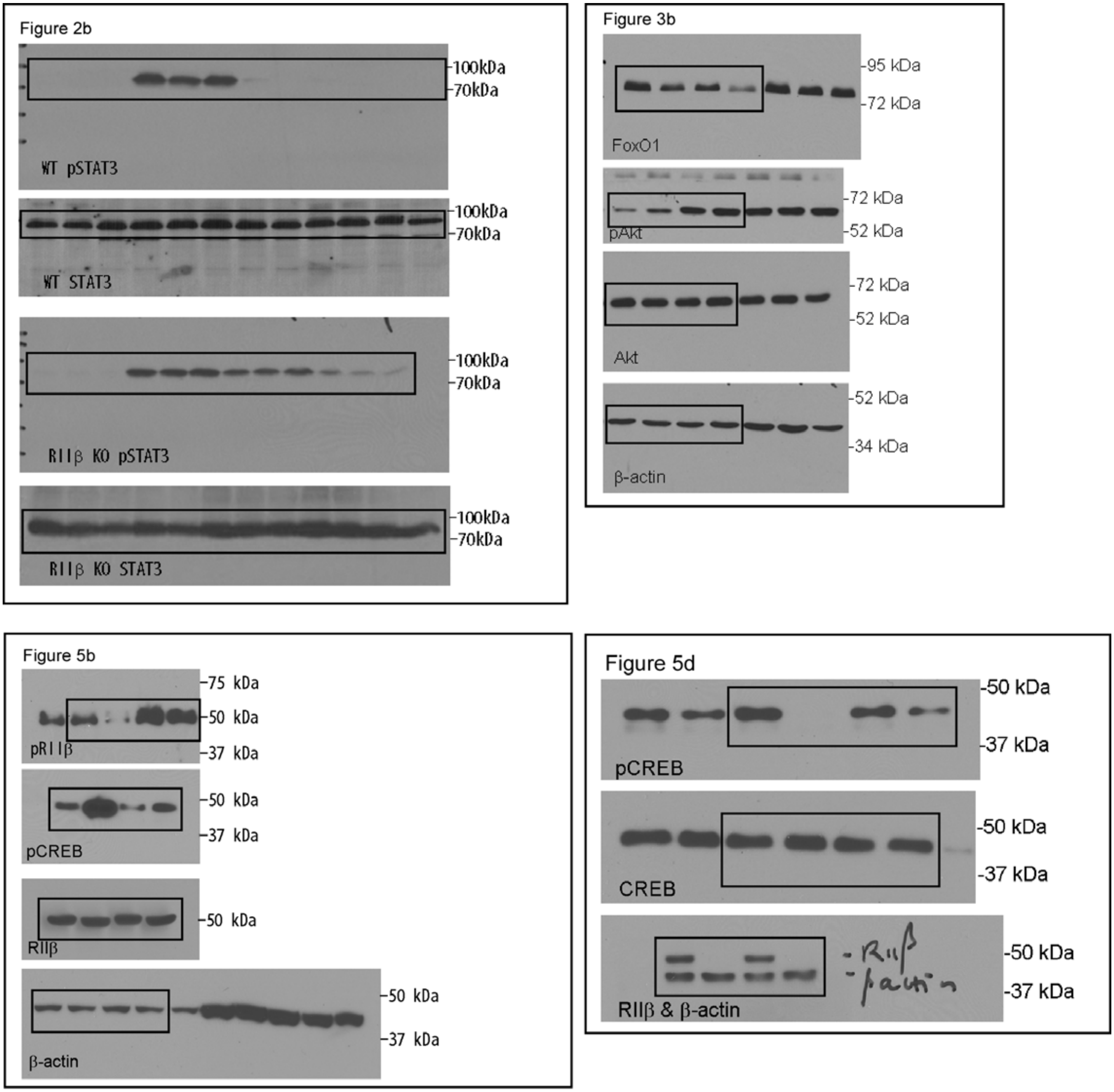

Supplementary Figure 2a

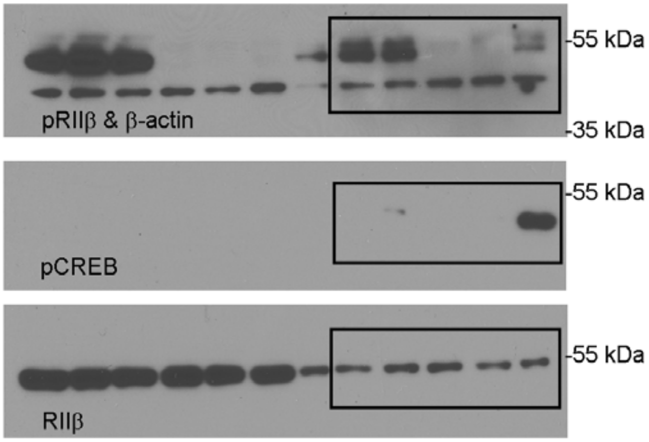

Supplementary Figure 2d

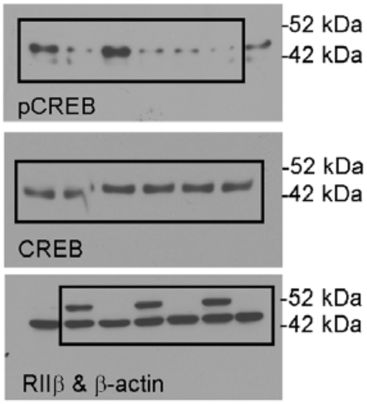

Supplementary Figure 2g

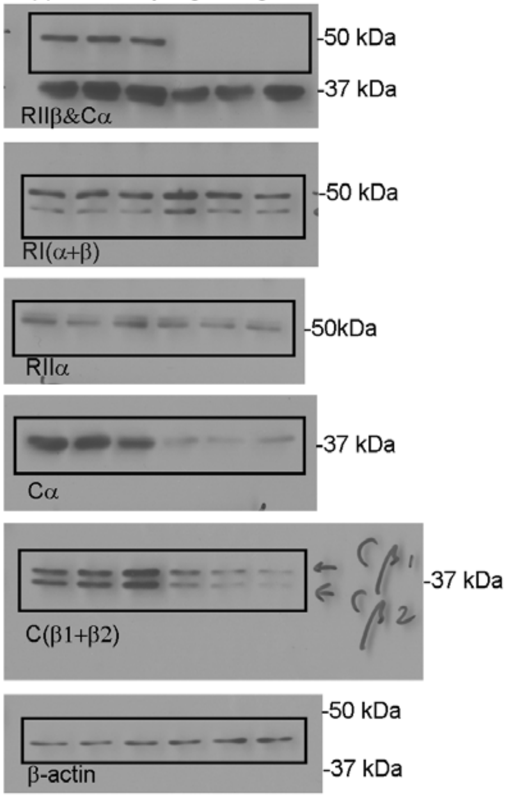

Supplementary Table 1 DNA primers for quantitative RT-PCR.

LepRb	Forward: 5’-TGTTTTGGGACGATGTTCCA-3’<br><br>Reverse: 5’-AAAGATGCTCAAATGTTTCAGGC-3’
Agrp	Forward: 5’-TAGATCCACAGAACCGCGAGT-3’<br><br>Reverse: 5’-GAAGCGGCAGTAGCACGTA-3’
Npy	Forward: 5’-CTCCGCTCTGCGACACTAC-3’<br><br>Reverse: 5’-AGGGTCTTCAAGCCTTGTTCT-3’
Pomc	Forward: 5’-CTGGAGACGCCCGTGTTTC-3’<br><br>Reverse: 5’-TGGACTCGGCTCTGGACTG -3’
Socs3	Forward: 5’-GAGTACCCCCAAGAGAGCTTACTA-3’<br><br>Reverse: 5’-CTCCTTAAAGTGGAGCATCATACTG-3’
FoxO1	Forward: 5’-CCCAGGCCGGAGTTTAACC-3’<br><br>Reverse: 5’-GTTGCTCATAAAGTCGGTGCT-3’
β-actin	Forward: 5’-AGTGTGACGTTGACATCCGTA-3’<br><br>Reverse: 5’-GCCAGAGCAGTAATCTCCTTCT-3’
Gapdh	Forward: 5’-AGGTCGGTGTGAACGGATTTG-3’<br><br>Reverse: 5’-TGTAGACCATGTAGTTGAGGTCA-3’
